# Zinc Oxide Nanoparticles for Revolutionizing Agriculture: Synthesis and Applications

**DOI:** 10.1155/2014/925494

**Published:** 2014-11-11

**Authors:** Sidra Sabir, Muhammad Arshad, Sunbal Khalil Chaudhari

**Affiliations:** Department of Botany, PMAS Arid Agriculture University Rawalpindi, Rawalpindi 46000, Pakistan

## Abstract

Nanotechnology is the most innovative field of 21st century. Extensive research is going on for commercializing nanoproducts throughout the world. Due to their unique properties, nanoparticles have gained considerable importance compared to bulk counterparts. Among other metal nanoparticles, zinc oxide nanoparticles are very much important due to their utilization in gas sensors, biosensors, cosmetics, drug-delivery systems, and so forth. Zinc oxide nanoparticles (ZnO NPs) also have remarkable optical, physical, and antimicrobial properties and therefore have great potential to enhance agriculture. As far as method of formation is concerned, ZnO NPs can be synthesized by several chemical methods such as precipitation method, vapor transport method, and hydrothermal process. The biogenic synthesis of ZnO NPs by using different plant extracts is also common nowadays. This green synthesis is quite safe and ecofriendly compared to chemical synthesis. This paper elaborates the synthesis, properties, and applications of zinc oxide nanoparticles.

## 1. Introduction

### 1.1. Nanotechnology

Nanotechnology is an emerging technology, which can lead to a new revolution in every field of science [1]. This technology is used with association to optics, electronics, and biomedical and materials sciences. Research in this field has gained momentum in the recent years by providing innovative solutions in different scientific disciplines.

Nanotechnology deals with nanoparticles that are atomic or molecular aggregates characterized by size less than 100 nm. These are actually modified form of basic elements derived by altering their atomic as well as molecular properties of elements [2, 3]. Nanoparticles gained considerable attraction because of their unusual and fascinating properties, with various applications, over their bulk counterparts.

### 1.2. Zinc Oxide Nanoparticles

Zinc oxide is an inorganic compound with the molecular formula ZnO. It appears as a white powder and is nearly insoluble in water. The powder ZnO is widely used as an additive in numerous materials and products including ceramics, glass, cement, rubber (e.g., car tyres), lubricants, paints, ointments, adhesives, plastics, sealants, pigments, foods (source of Zn nutrient), batteries, ferrites, and fire retardants. In the Earth crust ZnO is present as zincite mineral but mostly ZnO used for commercial purposes is produced synthetically. ZnO is often called II-VI semiconductor in materials science because zinc and oxygen belong to the 2nd and 6th groups of the periodic table. ZnO semiconductor has several unique properties such as good transparency, high electron mobility, wide band gap, and strong room temperature luminescence. These properties account for its applications in transparent electrodes in liquid crystal display and in energy-saving or heat-protecting windows and other electronic applications. Zinc oxide (wurtzite, p63m) is known as wide band gap semiconductor with band gap energy of 3.3 eV at room temperature (RT). Nowadays the unique properties of nanomaterials have motivated the researchers to develop many simpler and inexpensive techniques to produce nanostructures of technologically important materials. Several metal oxide nanoparticles are produced with possible future applications. Among them zinc oxide is considered to be one of the best exploited at nanodimensions. The wide band gap and large excitonic binding energy have made zinc oxide important both for scientific and industrial applications [4].

#### 1.2.1. Crystal Structure of ZnO

**Figure 1:**
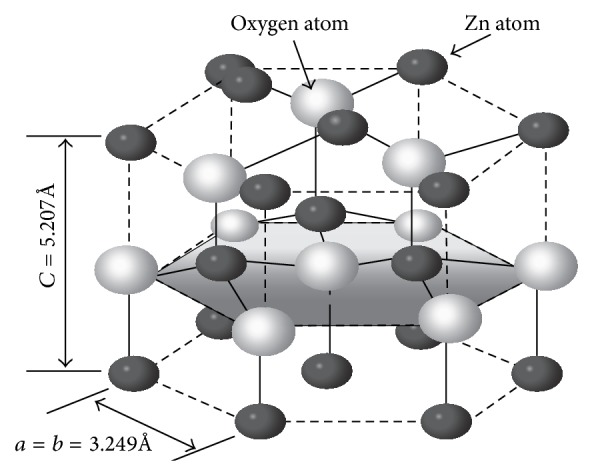
Tetrahedral structure of ZnO.

Crystalline ZnO has a wurtzite (B4) crystal structure, having a hexagonal unit cellwith two lattice parameters *a* and *c*. In this wurtzite hexagonal structure each anion is surrounded by four cations at the corners of the tetrahedron, which shows the tetrahedral coordination and hence exhibits the sp3 covalent bonding. The tetrahedral configuration of ZnO gives rise to a noncentrosymmetric structure [5–7] (Figure 1).

## 2. Chemical Synthesis of Zinc Oxide Nanoparticles

Nanomaterials or nanostructures can be synthesized by a variety of techniques such as spray pyrolysis, thermal decomposition, molecular beam epitaxy, chemical vapor deposition, and laser ablation.

### 2.1. Advantages of Chemical Synthesis

Chemical synthesis is one of the most important techniques which can be performed by using a range of precursors and different conditions like temperature, time, concentration of reactants, and so forth. Variation of these parameters leads to morphological differences in size and geometries of resulting nanoparticles.

Different chemical methods used for the synthesis of ZnO NPs are listed below.

### 2.2. Chemical Reaction of Zinc Metal with Alcohol

Mostly alcoholic media like ethanol, methanol, or propanol are used for chemical synthesis of ZnO nanoparticles. Typically in this synthesis 5 mg of zinc metal powder is added to 10 mL of ethanol. Further this reaction mixture is sonicated for 20 minutes and transferred into a stainless steel autoclave and sealed under inert conditions. The reaction mixture is heated slowly (2°C to 200°C per minute) and maintained at this temperature for 24 to 48 hours. The resulting suspension will then be centrifuged to retrieve the product, washed, and then finally vacuum dried. In alcoholic media growth of oxide particles is slow and controllable [8].

### 2.3. Vapor Transport Synthesis

The vapor transport process is most common method for the synthesis of ZnO nanostructures. In this process, zinc and oxygen or oxygen mixture vapors are transported and react with each other resulting in formation of ZnO nanostructures. There are numerous ways to generate Zn and oxygen vapor. Decomposition of ZnO is an easier, direct, and simple method; however, it is limited to very high temperatures such as ~1400°C.

Another direct method involves heating of zinc powder under flow of oxygen. It involves relative low growth temperature (500~700°C), but the ratio between the Zn vapor pressure and oxygen pressure must need to be carefully controlled in order to obtain the desired ZnO nanostructures. It has been observed that the change in this ratio results into a large variation in the morphology (size and geometry) of nanostructures [9].

### 2.4. Hydrothermal Technique

Hydrothermal technique is an efficient alternative synthetic method because of the low process temperature; it is much easy to control the particle size. This process has several advantages such as utilization of simple equipment, catalyst-free growth, low cost, uniform production, ecofriendliness, and being less hazardous over other growth processes. This method is attractive one for microelectronics and plastic electronics due to low reaction temperatures. This technique has been successfully employed for preparation of ZnO NPs and other luminescent materials. The particle morphology and size can be controlled through the hydrothermal process by adjusting the reaction temperature, time, and precursors concentration.

For synthesizing the ZnO nanoparticles, stock solutions of Zn (CH_3_COO)_2_ *·*2H_2_O (0.1 M) are prepared and then to this stock solution, 25 mL of NaOH (from 0.2 M to 0.5 M) solution prepared in methanol is added under stirring to get the pH value of reactants between 8 and 11. Further these solutions are transferred into Teflon lined sealed stainless steel autoclaves and maintained at various temperatures in the range of 100–200°C for 6 to 12 hours under autogenous pressure. The resulting white solid product will be washed with methanol, filtered, and dried in air in a laboratory oven at 60°C. Then the characterization of synthesized samples will be done to determine their structures by X-ray diffraction technique [10].

### 2.5. Precipitation Method

**Figure 2:**
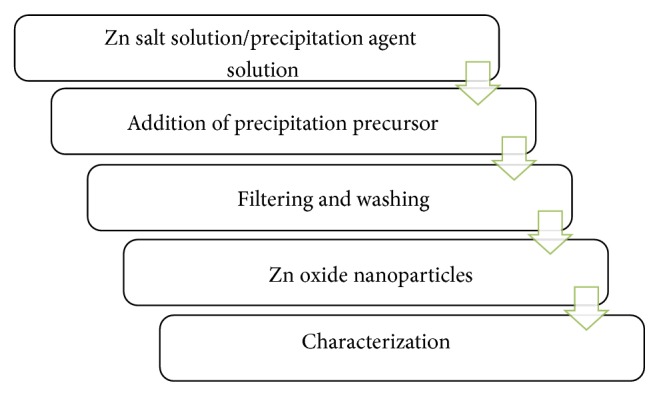
Precipitation method.

In this method ZnO can be synthesized by using zinc nitrate and urea as precursors. In a typical synthesis, 0.5 M (4.735 gm) zinc nitrate (Zn(NO_3_)_2_ *·*6H_2_O) is dissolved in 50 mL of distilled water and is kept under constant stirring for 30 minutes for complete dissolution. 1 M (3.002 gm) urea is also prepared in 50 mL of distilled water under constant stirring for 30 minutes; this urea solution acts as precipitating agent. This urea solution is added dropwise into zinc nitrate solution with vigorous stirring at 70°C for 2 hours to allow complete formation of nanoparticles. Finally precipitating solution turns whitish cloudy. This white precursor product is centrifuged at 8000 rpm for 10 minutes and washed with distilled water for the removal of any impurities or absorbed ions if present. Calcination of the obtained product will be done at 500°C in air atmosphere for 3 hr using muffle furnace [11]. All the chemical reactions which occurred in this process are shown in the flowchart (Figure 2).

### 2.6. Chemical Reaction of Zinc Acetate Dihydrate and NaOH

In this process 0.02 M aq. zinc acetate dihydrate is dissolved in 50 mL distilled water under vigorous stirring. Then aqueous 2.0 M NaOH is added drop by drop to reach pH 12 at room temperature; then whole solution is placed in a magnetic stirrer for 2 hours. After reaction is completed, then the obtained white precipitate is washed thoroughly with distilled water followed by ethanol to remove the impurities if present. Then precipitate will be dried in a hot air oven for overnight at 60°C and during drying the complete conversion of Zn(OH)_2_ into ZnO NPs takes place [12].

### 2.7. Disadvantages of Chemical Synthesis of Nanoparticles

The chemical synthesis methods of ZnO NPs like chemical precipitation, hydrothermal method, pyrolysis, chemical vapour deposition, and so forth result in the presence of some toxic chemicals adsorbed on the surface that may have adverse effects in medical applications. There are some reactions in these chemical procedures which require high temperature and high pressure for their initiation while some reactions require inert atmosphere protection or inert conditions. Some chemical techniques also involve utilization of certain toxic matters such as H_2_S, toxic template, and metallic precursors [13]. The chemicals used for synthesis of nanoparticles and for their stabilization are toxic and lead to nonecofriendly byproducts [14].

## 3. Green Synthesis of Zinc Oxide Nanoparticles

Green synthesis procedures involve the plant based synthesis of nanoparticles. Green synthesis techniques make use of somewhat pollutant-free chemicals for synthesis of nanostructures. It embraces the use of ecofriendly and safe solvents such as water, natural extracts.

So biological approaches using microorganisms and plants or plant extracts for synthesis of metal nanoparticles have been suggested as safe alternatives to chemical methods. In biogenic synthesis of nanoparticles, several biological systems including bacteria, fungi, and yeast have been used safely [15]. But synthesis of nanoparticles by using microorganisms is somewhat difficult because it involves elaborate process of maintaining cell cultures, intracellular synthesis, and multiple purification steps.

### 3.1. Advantages of Green Synthesis of Nanoparticles

In present times “green” method in the synthesis of nanoparticles has greatly become a topic of interest because the conventional chemical methods are expensive and require the use of chemical compounds/organic solvents as reducing agents which are toxic as well [16].

Green chemistry reduces pollution risk at source level and it is enhanced to prevent waste rather than treat or clean up waste after it is formed. The principle focuses on choice of reagents which are ecofriendly. Although physical and chemical methods are quick and easier for nanoparticles synthesis the biogenic technique is better and ecofriendly [17, 18].

### 3.2. By Using Leaf Extract of* Coriandrum sativum*

ZnO NPs can be synthesized by using the leaves extract of plant* Coriandrum sativum*. In this procedure, 50 mL of distilled water is taken and 0.02 M aqueous zinc acetate dihydrate is added into it under constant stirring. Then after 10 min stirring the aqueous leaf extract of* Coriandrum* is introduced at different sets (0.25, 0.5, 1 mL) into the above solution. 2.0 M NaOH is also added to make pH 12 resulting in a pale white aqueous solution. It is then placed in a magnetic stirrer for 2 hrs.

After stirring, pale white precipitate is taken out and washed many times with distilled water followed by ethanol to make it free from impurities. Then after drying at 60°C in vacuum oven overnight, a pale white powder of ZnO nanoparticles will be obtained [19, 20].

### 3.3. By Using Leaf Extract of* Calotropis gigantea*

The plant* Calotropis gigantea* has potential to be utilized for synthesis of ZnO NPs. 50 mL of* Calotropis gigantea* leaves extract is taken and boiled to 60–80 degree temperature by using a stirrer-heater or hot plate. Then 5 grams of zinc nitrate is added to the solution as the temperature reaches 60 degree Celsius. This whole mixture is then boiled till it reduces to a deep yellow color paste. This paste is then collected in a ceramic crucible and heated in furnace at 400°C for 2 hours. A light yellow color powder will be obtained and this is carefully collected and packed for further characterization purposes. The material is grounded in a mortar-pestle so as to get a finer nature product for characterization purposes [20].

### 3.4. By Using Leaf Extract of* Acalypha indica*

In this procedure first of all fresh leaves of* Acalypha indica* are taken and they are washed methodically with double distilled water and then grinded and the extracts are filtered through Whatman filter paper. Zinc acetate dihydrate (99% purity) and sodium hydroxide (pellet 99%) are utilized as the precursor material. Zinc acetate dihydrate is added to distilled water under vigorous stirring and after ten minutes stirring, the aqueous leaf extract of* Acalypha* is introduced into the above solution followed by the addition of aqueous 2.0 M NaOH; it results in a white aqueous solution at pH 12. The pH of the medium greatly influences the size of ZnO nanoparticles. Then the above solution will be positioned in a magnetic stirrer for 2 hours. Finally the precipitates are taken out and washed repetitively with distilled water followed by ethanol to remove the impurities of the obtained product. A white powder of ZnO nanoparticles will be obtained after drying at 60°C in vacuum oven overnight [12].

**Figure 3:**
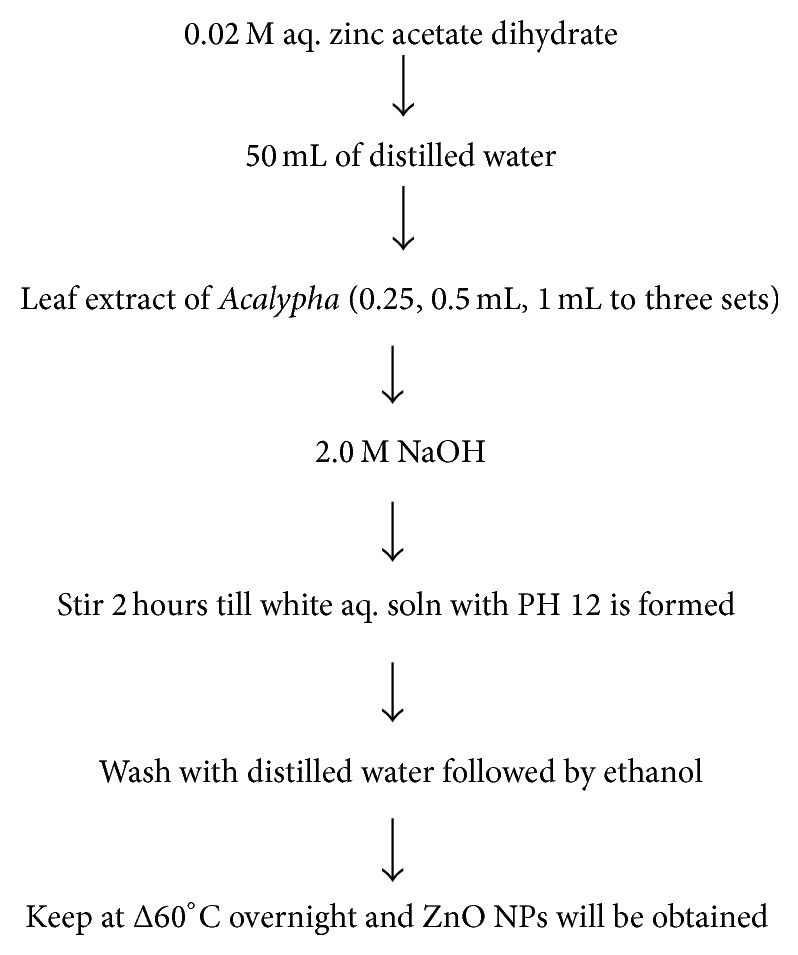


Schematic representation of this procedure is as in Figure 3.

### 3.5. By Using Milky Latex of* Calotropis procera*

Milky latex of plant* Calotropis procera* (AK, Maddar) is useful for synthesis of zinc oxide nanoparticles. In this procedure 0.02 M aq. solutions of zinc acetate dehydrate is mixed in 50 mL of distilled water under continuous stirring. After stirring for 10 minutes, latex of* Calotropis procera* 0.25, 0.5 mL, and 1.0 mL will be added in three sets into the above solution and after addition of milky latex, 2.0 M NaOH aqueous solution will be also introduced into the above aqueous solution; it will result in a white aqueous solution of pH 12, which is then placed on magnetic stirrer for continuous stirring for 2 hr. Finally the precipitate is taken out and washed with distilled water 2 or 3 times followed by ethanol also to remove the impurities present in the final product. Then a white powder will be obtained after drying it at 60°C in vacuum oven overnight [20].

### 3.6. By Using Rice as Soft Biotemplate

*Oryza sativa* rice is a renewable and abundant bioresource having unique characteristics that can be used as a biotemplate for the synthesis of various functional nanomaterials. The ZnO particles can be synthesized through hydrothermal-biotemplate method using zinc acetate, sodium hydroxide, and uncooked rice flour at various ratios used as precursors at 120°C for 18 hours. This biotemplate also influences morphology and size of ZnO NPs [21].

## 4. Distinguishing Properties of Zinc Oxide Nanoparticles

Zinc oxide nanoparticles possess the following distinguishing properties.

### 4.1. Physical Properties of ZnO NPs

Zinc oxide nanoparticles have tremendous physical properties. This is worth noting that as the dimension of semiconductor materials shrinks down continuously to nanometer or even smaller scale than in this reduction some of their physical properties undergo changes known as “quantum size effects.” For example, quantum confinement increases the band gap energy of quasi-one-dimensional (Q1D) ZnO, which has been confirmed by photoluminescence [22] (Table 1).

### 4.2. Optical Properties of ZnO NPs

Intrinsic optical properties of ZnO nanostructures are being intensively studied for implementing photonic devices. Photoluminescence (PL) spectra of ZnO nanostructures have been extensively reported, [23]. From the photoconductivity measurements of ZnO nanowires, it is found that the presence of O_2_ has an important effect on the photoresponse. It was found that the desorption-adsorption process of O_2_ affects the photoresponse of ZnO nanowire. Upon illumination, photogenerated holes discharge surface chemisorbed O_2_ through surface electron-hole recombination, while the photogenerated electrons significantly increase the conductivity. When illumination is switched off, O_2_ molecules readsorb onto nanowire surface and reduce the conductivity [24, 25].

### 4.3. Antimicrobial Properties of ZnO NPs

Antimicrobial activities of metal oxide (ZnO NPs) powders against* Staphylococcus aureus*,* Escherichia coli*, or fungi were quantitatively evaluated in culture media. It was observed that the growth inhibition was solely higher in biologically synthesized ZnO than chemical ZnO nanoparticle as well as other common antimicrobials. The enhanced bioactivity of these smaller particles is attributed to the higher surface area to volume ratio. The ZnO nanoparticles constitute an effective antimicrobial agent against pathogenic microorganisms. Basically the detected active oxygen species generated by these metal oxide particles could be the main mechanism of their antibacterial activity.

The antibacterial mechanism of ZnO NPs involves the direct interaction between ZnO nanoparticles and cell surfaces affecting cell membrane permeability; afterwards these nanoparticles enter and induce oxidative stress in bacterial cells, which results in the inhibition of cell growth and eventually cell death; the demonstrated antibacterial activity of ZnO NP recommends its possible application in the food preservation field. It can be applied as a potent sanitizing agent for disinfecting and sterilizing food industry equipment and containers against the attack and contamination with food borne pathogenic bacteria. The NPs of ZnO showed both toxicity on pathogenic bacteria (e.g.,* Escherichia coli* and* Staphylococcus aureus*) and beneficial effects on microbes, as* Pseudomonas putida*, which has bioremediation potential and is a strong root colonizer [26].

## 5. Applications and Uses of Zinc Oxide Nanoparticles

ZnO NPs have attracted intensive research efforts for their unique properties and versatile applications in transparent electronics, ultraviolet (UV) light emitters, piezoelectric devices, chemical sensors, and spin electronics [5, 27].

ZnO is nontoxic; it can be used as photocatalytic degradation materials of environmental pollutants. Bulk and thin films of ZnO have demonstrated high sensitivity for many toxic gases [28].

ZnO is currently listed as a “generally recognized as safe (GRAS)” material by the Food and Drug Administration and also used as food additive. ZnO nanostructures exhibit high catalytic efficiency, as well as strong adsorption ability, and are more frequently used in the manufacture of sunscreens. Most preferentially, among different metal oxide nanoparticles, zinc oxide (ZnO) nanoparticles have their own importance due to their vast area of applications, for example, gas sensor, biosensor, cosmetics, storage, optical devices, window materials for displays, solar cells, and drug-delivery [29–32].

### 5.1. Medicinal Uses of ZnO NPs

ZnO NPs play some potential role in CNS and perhaps during development processes of diseases through mediating neuronal excitability or even release of neurotransmitters. Some studies have indicated that ZnO NPs affected functions of different cells or tissues, biocompatibility, and neural tissue engineering [6, 33, 34] but little information is present about the influence on CNS and CNS related diseases. ZnO NPs have been suggested to modulate synaptic transmission in vitro and to change the spatial cognition capability via enhancing long-term potentiation (LTP) in rats. It is also suggested that exposure to ZnO NPs led to a genotoxic potential mediated by lipid peroxidation and oxidative stress [35, 36]. However, due to its targeting potential ZnO NPs have potential utility in the treatment of cancer and/or autoimmunity [37].

## 6. Role of ZnO NPs in Agriculture

Agriculture is backbone of third world economics but unfortunately now, the agriculture sector is facing various global challenges like climate changes, urbanization, sustainable use of resources, and environmental issues such as runoff, accumulation of pesticides and fertilizers; human population is increasing day by day and food demand is growing rapidly and estimated population increase in world from current level of 6 billion to 9 billion by 2050 is expected. So we must adopt efficient techniques to make agriculture more sustainable [38].

Nanotechnology has a dominant position in transforming agriculture and food production. Nanotechnology has a great potential to modify conventional agricultural practices. Most of the agrochemicals applied to the crops are lost and do not reach the target site due to several factors including leaching, drifting, hydrolysis, photolysis, and microbial degradation. Nanoparticles and nanocapsules provide an efficient means to distribute pesticides and fertilizers in a controlled fashion with high site specificity thus reducing collateral damage. Farm application of nanotechnology is gaining attention by efficient control and precise release of pesticides, herbicides, and fertilizers. Nanosensors development can help in determining the required amount of farm inputs such as fertilizers and pesticides. Nanosensors for pesticide residue detection offer high sensitivity, low detection limits, super selectivity, fast responses, and small sizes. They can also detect level of soil moisture and soil nutrients. Plants can rapidly absorb nanofertilizers. Nanoencapsulated slow release fertilizers can save fertilizer consumption and minimize environmental pollution.

Zinc oxide NPs have potential to boost the yield and growth of food crops. Peanut seeds were treated with different concentrations of zinc oxide nanoparticles. Zinc oxide nanoscale treatment (25 nm mean particle size) at 1000 ppm concentration was used which promoted seed germination, seedling vigor, and plant growth and these zinc oxide nanoparticles also proved to be effective in increasing stem and root growth in peanuts [39].

The colloidal solution of zinc oxide nanoparticles is used as fertilizer. This type of nanofertilizer plays an important role in agriculture. Nanofertilizer is a plant nutrient which is more than a fertilizer because it not only supplies nutrients for the plant but also revives the soil to an organic state without the harmful factors of chemical fertilizer. One of the advantages of nanofertilizers is that they can be used in very small amounts. An adult tree requires only 40–50 kg of fertilizer while an amount of 150 kg would be required for ordinary fertilizers. Nanopowders can be successfully used as fertilizers and pesticides as well [40, 41]. The yield of wheat plants grown from seeds which were treated with metal nanoparticles on average increased by 20–25% [42].

### 6.1. Impact of ZnO NPs on Plants

Nanoparticles of different metal oxides can play important role to promote the growth and yield of plants but now investigations about the toxicological effects of NPs continue to increase with time and only a few studies have been conducted to determine the effects of ZnO NPs on plants [43–46]. A research was carried out on seed germination and root growth of six higher plant species (radish, rape, ryegrass, lettuce, corn, and cucumber); the toxicity of five types of NPs (multiwalled carbon nanotubes, aluminum, alumina, zinc, and zinc oxide) showed that seed germination in general is not affected in most of the cases while root elongation was inhibited. The IC50 of ZnO NPs was estimated to be about 50 mg/L for radish and about 20 mg/L for rape and ryegrass [16].

Toxicological studies of ZnO nanoparticles on ryegrass show that, in the presence of ZnO NPs, the biomass of ryegrass was significantly reduced, root tip was shrunken, and root epidermal as well as cortical cells became highly vacuolated and collapsed. Mostly ZnO NPs remained adhered onto the surface of root and individual NPs observed to be present in the apoplast and protoplast of the root endodermis and stele as well. No or few or dissociated zinc ions were translocated in ryegrasses that are exposed to ZnO nanoparticles [44].

## 7. Negative or Toxic Impacts of ZnO NPs

Although the ZnO NPs have great commercial importance and are present in various commercial products there is clearly a growing public concern to know about the toxicological and environmental effects of ZnO NPs. Unfortunately, toxicological studies carried out on zinc oxide nanoparticles in the last ten years show that ZnO NPs have potential health as well as environmental risks. ZnO NPs can impose serious toxicity to bacteria,* Daphnia magna,* freshwater microalga, mice, and even human cells [36, 47–50].

### 7.1. Skin Penetration

ZnO NPs are particularly useful in sunscreens because they have intrinsic ability to filter ultraviolet UVA as well as UVB radiations. Due to this remarkable property they are providing broader protection than any other sun screening agent. But these nanoparticles have ability to penetrate to the skin and to reach viable cells resulting in the potential toxicity exerted by them. A comparative analysis of dermal penetration between different animal species was performed, rating them in the order rabbit > rat > pig > monkey > man and it is noted that pig and rat skin are up to 4 and 9–11 times more permeable than human skin, respectively. Overall, it is observed after many experiments that penetration through compromised skin was likely to be similar to normal skin but more work still needed to improve the understanding about this important safety issue [36, 50].

## 8. Conclusion

Nanotechnology is the emerging technology of present century operating in all fields of science. Zinc oxide nanoparticles stand out as one of the most versatile materials, due to their diverse properties, functionalities, and applications. ZnO NPs have tremendous physical and optical properties. They also possess antimicrobial actions against some bacteria and fungi. As far as synthesis of zinc oxide nanoparticles is concerned they can be synthesized by chemical methods but in recent times due to evolution of green chemistry, biogenic synthesis of ZnO NPs is also possible by using different plant extracts. The green synthesis of ZnO NPs is much safer and environment friendly compared to chemical synthesis because it does not lead to formation of toxic byproduct chemicals. As far as their usage is concerned nanoparticles play a significant role in agriculture, where colloidal solution of ZnO NPs is used in nanofertilizers. Application of these nanoparticles to crops increases their growth and yield. As food demand is increasing day by day the yield of staple food crops is much low. So it is need of the hour to commercialize metal nanoparticles for sustainable agriculture.

## Figures and Tables

**Table 1:** Physical properties of wurtzite zinc oxide nanoparticles [7].

Properties	Value
Density	5.606 g/cm^3^
Melting point	2248 K
Relative dielectric constant	8.66
